# THE STUDY OF MUSCLE, MOBILITY AND AGING (SOMMA): MITOCHONDRIAL ENERGETICS OF SKELETAL MUSCLE

**DOI:** 10.1093/geroni/igad104.2080

**Published:** 2023-12-21

**Authors:** Paul Coen, Russell Hepple, Bret Goodpaster

## Abstract

Aging leads to a decline in mitochondrial energetics of skeletal muscle. We hypothesize that worse mitochondrial energetics is associated with greater comorbidity burden and worse fatiguability. Furthermore, sex differences in mitochondrial decline with aging are poorly understood. Prior human muscle biopsy studies have tended to be small and did not account for confounding factors including physical activity and adiposity levels. The Study of Muscle, Mobility, and Aging (SOMMA) is an observational longitudinal cohort study of 879 older adults designed to discover the biological basis of mobility disability and to collect tissues and measurements for future studies of the biology of human aging. The multidisciplinary SOMMA team includes two clinical centers (University of Pittsburgh and Wake Forest University), a biorepository (Translational Research Institute at AdventHealth), and the San Francisco Coordinating Center (California Pacific Medical Center Research Institute). At baseline, SOMMA collected biospecimens (muscle and adipose tissue, blood, urine, fecal samples); a variety of questionnaires; physical and cognitive assessments; whole-body imaging (magnetic resonance and computed tomography); accelerometry; and cardiopulmonary exercise testing. Primary outcomes include change in walking speed and change in fitness (VO2 peak). In this symposium, we will present new findings from SOMMA baseline data that link skeletal muscle mitochondrial energetics to age-associated comorbidities, including diabetes, and performance fatiguability. We also highlight the critical role that sex differences in mitochondrial energetics plays in mobility limitation. SOMMA is supported by the National Institutes of Aging (R01AG059416).

